# Editorial: The Medial Prefrontal Cortex and Integration in ASD and Typical Cognition

**DOI:** 10.3389/fnhum.2019.00074

**Published:** 2019-03-05

**Authors:** Dorit Ben Shalom, Yoram S. Bonneh

**Affiliations:** ^1^Zlotowski Center for Neuroscience, Ben-Gurion University of the Negev, Beersheba, Israel; ^2^School of Optometry and Vision Science, Faculty of Life Sciences, Bar-Ilan University, Ramat Gan, Israel

**Keywords:** emotion, memory, sensory, motor, prefrontal cortex

A long standing theme in the study of ASD is the lack of, or atypicality, of cognitive integration. Despite its intuitive power, there have been very few attempts to specify the specific type of integration, that would allow for a systematic investigation of its neural basis. A recent paper in the Neuroscientist has attempted to do just that, in terms of a connection between subareas of the medial prefrontal cortex and the integration of familiar cognitive objects (albeit often under different names): perceptual objects, memory episodes, emotional states, and motor actions. The model suggested that in each of these four domains, three levels of processing can be identified: a basic pre-integrative level, an integrative level which produces the above mentioned cognitive objects, and a “logical” or higher-order level that performs selection/inhibition and perhaps combination on these basic type of objects. It further suggests that a lack of or atypicality of these objects is the source of some core difficulties in ASD.

This Research Topic explores within-discipline and across-discipline implications of the Ben Shalom (2009) model. The 12 papers hail from psychology, neuroscience, psychiatry, philosophy, and biology.

Broadly speaking, this Research Topic is divided into two sections: one that explores within-discipline implications of the model, and one that explores across-discipline connections.

This Research Topic includes 7 papers discussing the 4 cognitive domains contained in the model: emotion, memory, sensory-perceptual, and motor. There are 2 papers about each, apart from motor, probably reflecting our relative lack of knowledge about the processing of this domain in ASD.

Our initial hope was to have, within each domain, one paper that talks about difficulties in the integration *within* objects, and one about difficulties in the integration *between* objects, because we believe we see these two types of difficulties in different subgroups in the ASD population. This hope was partially fulfilled.

In the *emotion* domain, South and Rodgers mention alexithymia, which, although comorbid to ASD (e.g., Bird et al., 2010), is probably always present (although can be compensated for) in the subgroup with difficulties integrating typical *emotional states*, because these atypical states are inherently harder to identify and label.

The Komeda paper discusses how, based on mutual understanding, individuals with ASD respond empathically to others with these disorders. Being a high-level social cognition paper, it necessarily involves integration *between* emotional states, rather than the integration *within* the emotional states themselves.

In the *memory* domain, Lecouvey et al. talk about the ability to create a unified representation of the individual features of an event, i.e., the integration *within* memory events.

The Brezis paper discusses memory integration in the autobiographical narratives of individuals with autism. Being a high-level autobiographical narratives paper, necessarily involves integration *between* memory events, rather than the integration *within* the memory events themselves.

In the sensory domain, Smith et al. discusses the influence of both low-level features and information from meaningful context on the final percept, so in this sense it is about the integration *within* sensory objects.

The Martínez-Sanchis paper discusses difficulties in multisensory integration in people with autism spectrum disorders. Being a high-level multisensory integration paper, it necessarily involves integration *between* sensory objects, rather than *within* the sensory objects themselves.

In the motor domain, Fukui et al. discusses difficulties in the chaining of motor acts in adolescents and young adults with autism spectrum disorder. Being a high-level chaining paper, it necessarily involves integration *between* motor actions, rather than *within* the motor actions themselves.

The between-discipline section includes 5 papers, which are intended to connect the current functional neuroanatomical work with other disciplines.

The first paper, by Yao et al. is intended as a connection to *other* areas of cognitive neuroscience, specifically functional connectivity. It showed decreased functional connectivity between the precuneus/posterior cingulate gyrus and the medial prefrontal cortex in the default mode network.

The second paper, by Tzur, is intended as a connection to *psychiatry*. Perhaps most notably, it manages to establish a connection between Ben Shalom's (2009) model of the medial prefrontal cortex and integration in ASD and Schore (2013) regulation theory in which mother-infant mutual interpersonal emotion-regulation processes help the infant develop holistically integrated regulatory processes, that are supported by the prefrontal cortex.

The third paper, by Ferguson and Gao, is intended as a connection to *biology*, discussing the development of the thalamocortical connections between mediodorsal thalamus and prefrontal cortex, and its implication in cognition. Perhaps most notable in the present context, following just days behind the arrival and subsequent increase in the density of mediodorsal afferent terminals, the medial prefrontal cortex undergoes a vast increase in volume further suggesting that the mediodorsal thalamus plays a critical regulatory role over prefrontal cortical development.

The fourth paper, by Yatziv and Jacobson, is intended as a connection to *philosophy*. Specifically, they argue against the view that autistic subjects have a deficiency in the most basic form of perceptual consciousness namely, phenomenal consciousness. Instead, they maintain, the perceptual atypicality of individuals with autism is of a more conceptual and cognitive sort. Thus, the basic experiences of individuals with autism are likely to be similar to typical subjects experiences; the main difference lies in the sort of cognitive access the subjects have to their experiences, specifically the _*integration*_ of perceptual objects and concepts.

The fifth paper, by Ronel, is intended as a connection to *neurology*. It is the lateral prefrontal counterpart of Ben Shalom (2009), which suggested that the medial prefrontal cortex (including the medial orbital prefrontal cortex) is involved in the *integration* of perceptual objects (medial BA 11), memory episodes (medial BA 10), emotional states (medial BA9), and motor actions (medial BA 8). The current paper suggests that the corresponding *lateral* prefrontal areas (including the lateral orbital prefrontal cortex), re involved in the *selection/inhibition* of the same cognitive objects: perceptual objects (lateral BA 11), memory episodes (lateral BA 10), emotional states (lateral BA9), and motor actions (lateral BA 8).

At the core functional neuroanatomical level, these papers suggest a full model of the prefrontal cortex in terms of 4 streams of information (from ventral to dorsal): perceptual, memory, emotion, and motor. Within each stream, the medial prefrontal cortex is predicted to perform the *integration* of the associated cognitive objects, and the lateral prefrontal cortex their *selection/inhibition* (and perhaps also combination). On a more functional level, these papers go a long way toward describing how atypicality of medial prefrontal integration contributes to behavioral difficulties in ASD (such as agnosias, episodic memory difficulties, alexithymia, and dyspraxia). What is less clear, perhaps because the medial prefrontal cortex does not, in general, support any type of introspection *on its own workings*, is how this integration functions in typical cognition: in object recognition, in the creation of memory episodes, in the integration of one's own and perhaps others' emotional states, or in the construction of smooth, integrated motor actions. In that sense, the medial prefrontal cortex is perhaps the ultimate “cognitive black box”, as it shapes our own reality, and as such, should be a prime target for future work in both the neurosciences and cognitive psychology.

## Author Contributions

All authors listed have made a substantial, direct and intellectual contribution to the work, and approved it for publication.

### Conflict of Interest Statement

The authors declare that the research was conducted in the absence of any commercial or financial relationships that could be construed as a potential conflict of interest.
